# Novel budding mode in *Polyandrocarpa zorritensis*: a model for comparative studies on asexual development and whole body regeneration

**DOI:** 10.1186/s13227-019-0121-x

**Published:** 2019-04-03

**Authors:** Marta Scelzo, Alexandre Alié, Sophie Pagnotta, Camille Lejeune, Pauline Henry, Laurent Gilletta, Laurel S. Hiebert, Francesco Mastrototaro, Stefano Tiozzo

**Affiliations:** 1 CNRS, Laboratoire de Biologie du Développement de Villefranche-sur-Mer (LBDV), Sorbonne Université, 06230 Villefranche-sur-Mer, France; 2 Centre Commun de Microscopie Appliquée, UFR Sciences, Faculté des Sciences del’Université de Nice - Sophia Antipolis, 06108 Nice, France; 3 Departamento de Zoologia, Instituto Biociências, Universidade de São Paulo, São Paulo, 05508-090 Brazil; 4 Department of Biology and CoNISMa LRU, University of Bari Aldo Moro, 70125 Bari, Italy

**Keywords:** Ascidian, Evolution, Non-embryonic development, Tunicate, Vasal budding

## Abstract

**Background:**

In tunicates, the capacity to build an adult body via non-embryonic development (NED), i.e., asexual budding and whole body regeneration, has been gained or lost several times across the whole subphylum. A recent phylogeny of the family Styelidae revealed an independent acquisition of NED in the colonial species *Polyandrocarpa zorritensis* and highlighted a novel budding mode. In this paper, we provide the first detailed characterization of the asexual life cycle of *P. zorritensis*.

**Results:**

Bud formation occurs along a tubular protrusion of the adult epidermis, the stolon, in a vascularized area defined as budding nest. The bud arises through a folding of the epithelia of the stolon with the contribution of undifferentiated mesenchymal cells. This previously unreported mode of bud onset leads to the formation of a double vesicle, which starts to develop into a zooid through morphogenetic mechanisms common to other Styelidae. The budding nest can also continue to accumulate nutrients and develop into a round-shaped structure, designated as spherule, which represents a dormant form able to survive low temperatures.

**Conclusions:**

To understand the mechanisms of NED and their evolution, it is fundamental to start from a robust phylogenetic framework in order to select relevant species to compare. The anatomical description of *P. zorritensis* NED provides the foundation for future comparative studies on plasticity of budding and regeneration in tunicates.

**Electronic supplementary material:**

The online version of this article (10.1186/s13227-019-0121-x) contains supplementary material, which is available to authorized users.

## Introduction

In addition to sexual reproduction and embryogenesis, a significant number of metazoan species are able to propagate asexually or regenerate completely and form new bodies through non-embryonic development (NED) [1–3]. Many species of tunicates, the sister group of vertebrates [4], are capable of asexual development and whole body regeneration [5]. Tunicates include solitary species, which can only reproduce sexually, and colonial species that, in addition to sexual reproduction, are also able to propagate asexually and have extensive regenerative capabilities [5–7]. In tunicates, asexual reproduction and whole body regeneration occur by clonal replication whereby somatic tissues undergo a budding process that leads to the development of clonally related zooids. Current hypotheses for the phylogeny of the tunicates suggest many convergent acquisitions of NED across the whole subphylum [8–10], a view further supported by the diversity of tunicate budding modes. In fact, mechanisms of asexual development differ substantially among colonial species and involve differences in the body region where new buds appear, and differences in the types of cells and tissues that contribute to the development of new zooids [11–15]. Diversity in budding mode likely contributes to the wide variation in colony organization and integration among zooids found across the subphylum [16].

**Fig. 1:**
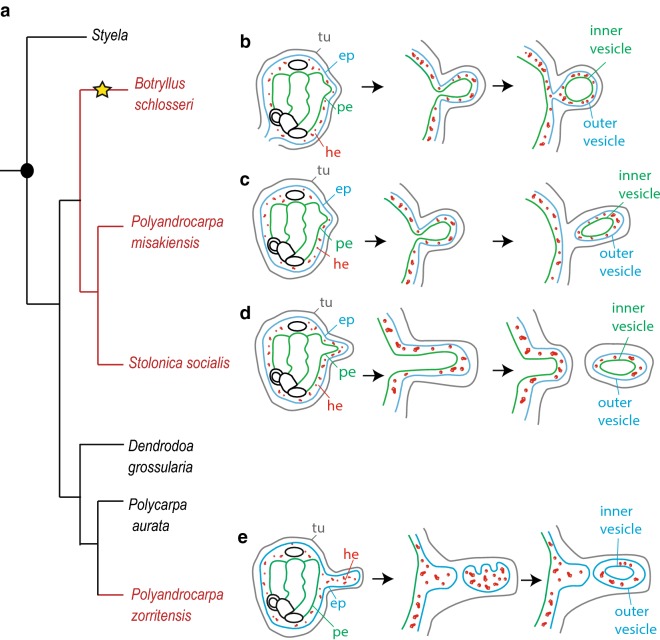
Phylogeny of the family Styelidae and diversity of budding modes. **a** Simplified phylogenetic tree of Styelidae family (modify from Alié et al. 2018), showing the distribution of solitary species (shown in black) and colonial species (in red). The yellow star indicates the branches in which vascular budding evolved. **b**–**d** Schematic representation of peribranchial budding in *Botryllus schlosseri* (**b**), *Polyandrocarpa misakiensis* (**c**), and *Stolonica socialis* (**d**), from peribranchial evagination to double vesicle stage. **e** Schematic representation of the budding process in *Polyandrocarpa zorritensis* from the formation of vascular stolon to double vesicle stage. (*tu* tunic, *ep* epidermis, *he* haemocytes, *pe* peribranchial epithelium)

In order to dissect the mechanisms of budding in ascidians and to understand the acquisition and loss of complex characters such as asexual propagation and whole body regeneration, it is necessary to broaden our knowledge of budding modes among different species, and to compare them in a phylogenetic perspective relying on robust trees. Most of our current knowledge of tunicate NED comes from a limited range of species. One of the most well-studied colonial ascidians belongs to the family Styelidae, *Botryllus schlosseri* (Pallas 1776). In this species, the bud originates from an evagination and thickening of two epithelia, the peribranchial epithelium and the surrounding epidermis, with the possible participation of mesenchymal cells [17]. The evagination arches progressively to form a double vesicle (Fig. 1): the outer vesicle derives from the adult epidermis and gives rise to the epidermis of the new zooid, while the inner vesicle derives from the peribranchial epithelium and undergoes morphogenetic changes that eventually give rise to the internal organs of the functional new zooid. Circulating mesenchymal cells aggregate within the folding epithelium and proliferate, participating in the formation of the organs [18–20]. The resulting bud remains attached to the colony by a short peduncle. This mode of reproduction, called peribranchial budding or blastogenesis, permits colonial growth and periodic zooidal renewal. In the family Styelidae, peribranchial budding is the main form of asexual development, with some minor variations between species (Fig. 1). For instance, in *Polyandrocarpa misakiensis* [21], mesenchymal cells are thought to transdifferentiate and integrate into the inner vesicle and the evaginated bud separates from the parent [22, 23]. In *Stolonica socialis* (Hartmeyer 1903), the atrial epithelium and epidermis protrude and extend considerably from the zooid body forming a process called a stolon [24]. When the tip of the stolon reaches a certain mass, it is constricted off forming a bud disconnected from the maternal zooid [25].

In a recent phylogenetic reconstruction of the family Styelidae, we revealed that all the species undergoing peribranchial budding belong to a single clade (Fig. 1, from [10]). Within this clade, the genera *Botryllus*, *Botrylloides,* and *Symplegma* are also able to regenerate via a second budding mode, vascular budding, in which the bud originates from the aggregation of circulating mesenchymal cells interacting with the vascular epithelium [26, 27]. The above-cited phylogeny of Styelidae also revealed that the colonial species *Polyandrocarpa zorritensis* (Van Name 1931) does not group with the clade of “peribranchial budders” but is rather more closely related to solitary styelid species (Fig. 1). These results suggest a convergent acquisition of NED in *P. zorritensis*, and prompted us to further describe this process [10, 28]. Preliminary histological analyses revealed that, as in other colonial styelids, a double-layered vesicle is formed prior to bud organogenesis [10]. However, in *P. zorritensis*, the inner vesicle does not derive from peribranchial epithelium, but it originates from the epidermis of the circulatory system, which has ectodermal embryonic origin [7]. This undescribed mode of budding not only supports the independent acquisition of coloniality and makes *P. zorritensis* a key species for comparative studies aiming to understand the distribution of coloniality in the group, but also opens up new territory to explore the developmental mechanisms behind the capacity for budding.

In this paper, we provide the first detailed characterization of the asexual life cycle of *Polyandrocarpa zorritensis* and we describe the ontogenesis of this unreported form of ascidian budding that we name “vasal budding.”

## Materials and methods

### Sample collection and specimen husbandry

Colonies of *Polyandrocarpa zorritensis* were collected in the harbors of Taranto (Mare Piccolo, 40°28′48.29″N 17°16′4.33″E) and La Spezia (Assonautica Benedetti, 44°06′10.7″N 9°49′34.5″E) (Italy). Colonies in the field are composed of feeding zooids and often a layer of smaller orange globular non-feeding structures that we have termed *spherules.* The spherules were gently separated from the colony and stored in circulating sea water system at 11 °C (at salinity of 39–40‰). New zooids were grown by transferring the spherules to glass microscope slides at 24 °C. We use the term “hatching” for the emerging of a new zooid from a spherule. The animals were maintained at 24 °C in open sea water systems and fed with a mix of living algae (*Tisochrysis lutea*) and concentrated algae (Isochrysis 100 and Shellfish Diet 1800, Red Maricolture Inc).

### Light and transmission electron microscopy (TEM)

Inclusion of *Polyandrocarpa zorritensis* specimens for paraffin sectioning and hematoxylin/eosin staining were performed as described in Alié et al. [10]. Samples for semi-thin and ultra-thin sectioning were fixed with a 3% solution of glutaraldehyde in sodium cacodylate buffer (pH 7.3), post-fixed with osmium tetroxide (OsO_4_) 1% cacodylate buffer, dehydrated using acetone, and embedded in epoxy resin. An UltracutE Reichert ultramicrotome was used for the sectioning. Semi-thin sections (1–2 µm) were stained with a solution of methylene blue 1% in sodium tetraborate + azur II 1% in water (v/v), and images were analyzed on a ZEISS microscope Axio ImagerA2. Ultra-thin sections (60–80 nm) were contrasted with uranyl acetate and lead citrate and observed under a transmission electron microscope TEM JEM 1400 JEOL, and imaged using a MORADA SIS camera (Olympus).

### Effect of low-temperature storage and spherule size on hatching and growth

To test whether storing the spherules at 11 °C had any negative effect on the hatching rate, two different batches of spherules were used. The first corresponds to spherules collected in Spring 2016 (April) and directly transferred to glass slides at 24 °C in the laboratory. The second batch corresponds to spherules that were kept at 11 °C in circulating sea water for 7 months before being transferred to 24 °C. After 7 days, the number of the zooids that were generated in relation to the number of spherules sowed was counted. After 13 days, the number of zooids bearing stolons was counted.

In the following experiments, the size of zooids and budding nests (a term we use for newly formed pre-buds made of vascular ampullae aggregations) was estimated photometrically, by delineating their periphery manually and measuring the area using ImageJ software [29]. To test the effect of spherule size, we arbitrarily split 45 spherules into three categories of size (“small” from 1 to 1.5 mm in diameter; “medium” from 1.5 to 2 mm in diameter; “big” 2 mm in diameter and larger) and then monitored the size of zooids derived from these spherules, considering also the number of stolons born by each zooid. Statistical significance was assessed using a Mann–Whitney test. To test the effect of budding nest size, 32 budding nests were followed during 6 days post-abscission (i.e., separation of the bud from its parental zooid by cutting the stolonial vessel with a razor blade) and the size of zooids was then measured. A linear regression curve was drawn using Excel software.

## Results

### Asexual life cycle of *P. zorritensis* in captivity

**Fig. 2:**
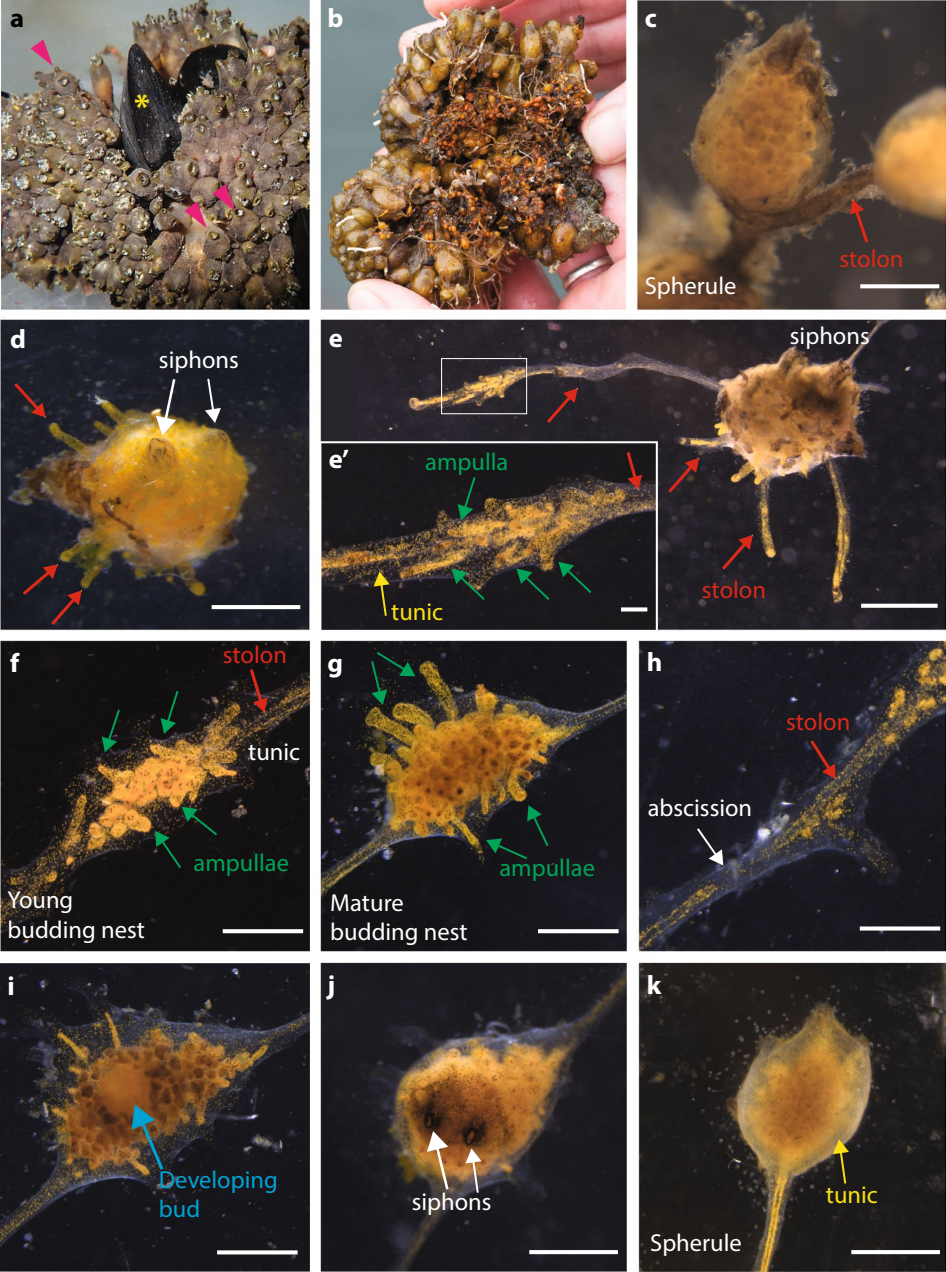
Asexual cycle of *Polyandrocarpa zorritensis* in captivity. **a** *Polyandrocarpa zorritensis* colony. Adult zooids can be found on other animals, such as mussels (yellow asterisk). Pink arrowheads show examples of individual zooids. **b** Bottom side of the same colony as in (**a**), showing the dense network of stolons and spherules. **c** Detail of a spherule and the stolon (red arrow) connecting the spherule to the rest of the colony. **d** One spherule one week after being transferred at 24 °C. The two siphons (white arrows) are open and the protrusions (red arrows) that will attach to the substrate are recognizable. **e** One completely developed zooid with several stolons (red arrows). **e’** Close-up view of one stolon. It is possible to recognize the main blood vessel (red arrow) and the ramified ampullae connected to it (green arrows), oriented in the same direction as the main vessel. The tunic (yellow arrow) covers the whole structure. **f** Young budding nest. The ampullae (green arrows) increased in number along the vessel (red arrow). **g** Mature budding nest, composed of more compact ampullae (green arrows) that form a dome, highly pigmented on the top. **h** Detail of a vessel at the abscission site (white arrow). **i** Bud (blue arrow) developing inside the nest after abscission. **j** Newly budded zooid with open siphons (white arrows). **k** Spherule obtained by transferring a budding nest from 24 °C to 11 °C. (Scale bars **c**–**e**: 1 mm; **e’**: 100 μm; **f**–**k**: 1 mm)

The budding process in *Polyandrocarpa zorritensis* has been briefly described by Brunetti and Mastrototaro [28] and by Alié et al. [10]; however, detailed descriptions of the colonial life cycle and bud histology are still missing. Wild colonies are composed of a few hundred zooids clustered together and connected by a dense network of orange-colored stolons covered by a thick tunic (Fig. 2a, b). Most of these stolons have the shape of a pearl necklace, with spherical structures of 1 to 2.5 mm in diameter connected to each other by thin segments of stolon (Fig. 2c). Each of these spherules can be easily separated from the colony—their stiffness permits their manipulation without damage—and stored at 11 °C for up to several months without any detectable morphological alteration.

When transferred to glass slides at 24 °C, the spherules show morphological changes and eventually give rise to zooids. After 1 to 5 days, the spherule loses its round shape, becomes more elongated, and shows lighter orange coloration (Additional file 1: Figure S1); after 6 to 10 days two open siphons become visible (Fig. 2d) indicating that filter feeding activity has initiated. Up to 12 months at 11 °C, spherules are still able to “hatch” and develop into filtering zooids, although this process is slightly slower than freshly collected ones (Additional file 2: Figure S2). When a colony is placed at 11 °C, most of the zooids regress and die, while only the spherules remain unaffected.

During the development from spherule to zooid, the body surface sprouts several stolons along which buds will appear. The stolons begin as small protrusions that allow the attachment of the zooid to the substrate (Fig. 2d). Usually, one or several of these body protrusions keep growing producing elongated stolons running along the substrate (Fig. 2e). The size of a zooid as well as the number of the emitted stolons is correlated to the size of the spherule from which it develops (Additional file 3: Figure S3). The stolon consists of a monolayered epidermal vessel covered with a thin layer of tunic. Circulating mesenchymal cells, hemocytes, flow through the stolonal lumen switching direction periodically (Additional file 4: Movie 1). Unlike the stolons of other ascidian species [30, 31], no central septum is present in the lumen of the *P. zorritensis* stolon. Along the whole stolon, anastomosed oval structures named ampullae grow parallel to the central vessel (Fig. 2e–e’). In some points along the stolon, the number of ampullae increases and their orientation changes, forming a tridimensional highly pigmented dome-like cluster, here named “budding nest” (Fig. 2f, g). Bud development begins at the center of these structures. Along a single stolon, it is possible to observe several budding nests, which emerge without any clear temporal or spatial order, but rather form upon substrate irregularities such as the angle of a Petri dish or scratches on a glass slide (personal observation).

Bud development is triggered upon complete isolation of the budding nest from the colony, which occurs spontaneously by abscission of the stolonial epidermis and consequent interruption of hemocyte circulation between the colony and the nest (Fig. 2h). Under laboratory conditions, budding can be triggered by cutting the stolon with a razor blade. About 24 h after abscission, the first signs of budding become externally visible: the number of ampullae decreases and a new zooid grows in the center of the nest (Fig. 2i). Within 4–5 days after the abscission, all the ampullae have regressed and two siphons open at the top of the newly formed zooid (Fig. 2j), while the axis defined by the endostyle is always perpendicular to the substrate, the axis along the two siphons is established randomly in respect to the orientation of the stolon. Just as we observed with spherules, the size of a zooid is proportional to the size of the nest from which it comes (Additional file 5: Figure S4). If abscission does not occur, the budding nest keeps growing, and if transferred at 11 °C, it eventually transforms into a dormant spherule (Fig. 2k).

### The origin of budding: formation of the budding nest and the epidermal accumulation of reserve nutrients

**Fig. 3:**
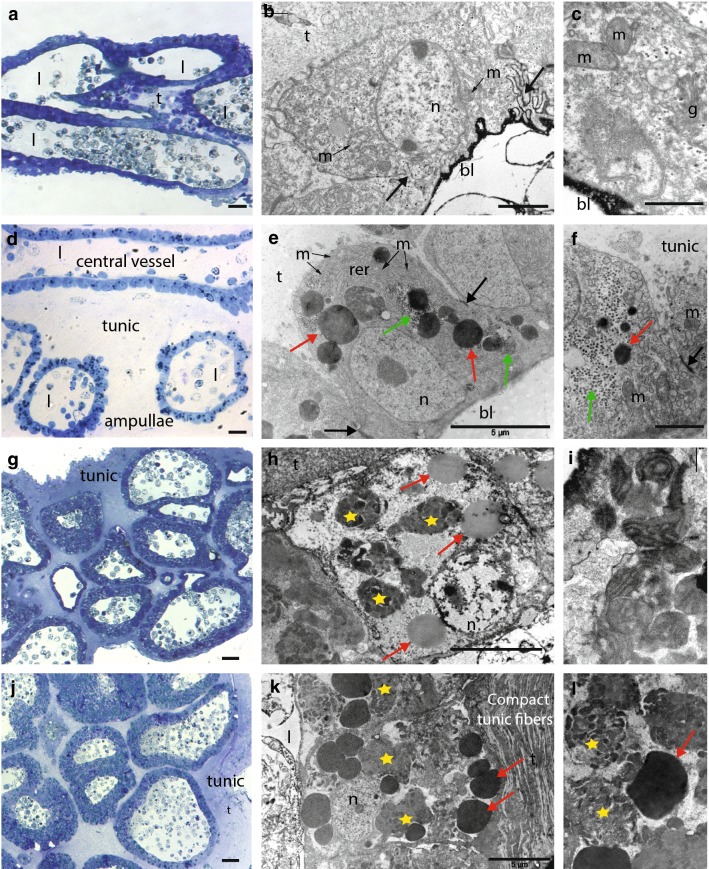
Histological and ultra-structural characterization of the stolon, budding nest, and spherule. **a** Transversal semi-thin section of a stolon. **b** A stolonial epithelial cell, with strongly electron-dense basal lamina (bl) and several mitochondria (m). The cells are connected by elaborated intercellular contacts (black arrows). **c** Detail of a stolonial epithelial cell, showing Golgi apparatus (g), basal lamina (bl), and mitochondria (m). **d** Longitudinal semi-thin section of a young nest. **e** Epithelial cells of the stolonial vessel, containing extensive rough endoplasmic reticulum (RER), large electron-dense granules (red arrows), and glycogen granules (green arrows). Tight junctions (black arrows) connect the neighboring cells. **f** Detail showing the intercellular junctions (black arrow), some small electron-dense granules (red arrow) and glycogen granules (green arrow). **g** Semi-thin sections of a mature nest. **h** Epithelial cell from a budding nest, containing spherical electron-dense granules (red arrow) and heterogeneous inclusions (yellow star). **i** Detail of the inclusions’ content. **j** Semi-thin section of a spherule. **k** An epithelial cell from a spherule, completely filled by heterogeneous inclusions (yellow stars) and electron-dense granules (red arrows). **l** Detail showing the two different types of inclusions present in the epithelial cells. (Scale bars. Semi-thin sections: 10 µm; **b**: 2 µm; **c**: 1 µm; **e**: um; **f**: 2 µm; **h**: 5 µm; **i**: 1 µm; **k**: 5 µm; **l**; 5 µm) (*bl* basal lamina, *g* Golgi, *l* vessel lumen, *m* mitochondria, *n* nucleus, *rer* rough endoplasmic reticulum, *t* tunic)

During formation of a budding nest, the epidermis of the stolonial vessel and its surrounding ampullae undergo drastic histological and cellular changes. Histological sections confirmed the absence of a mesenchymal septum in the stolon. In the growing stolon, before nest formation, the stolonial wall is constituted by a monolayered epidermis composed of flat cells of 6–7 µm, with the highly electron-dense basal lamina facing the vascular lumen, while the apical side is covered with a layer of tunic 5–10 µm thick (Fig. 3a–c). Cells contain extensive RER (rough endoplasmic reticulum) and an abundant Golgi apparatus (Fig. 3c). At the basal pole, elaborated intercellular contacts connect the nearby cells (Fig. 3b, black arrows). The continuity with the zooid epidermis and the orientation of the epithelial cells confirm the epidermal nature of the stolon rather than peribranchial expansion as in other styelids (e.g., *Stolonica socialis*, [25]) (Fig. 3a).

When a budding nest starts forming, the tunic thickens (to around 100 µm) and ramifying ampullae accumulate around the central vessel (Figs. 2f, 3d). Epithelial cells of both the central vessel and the ampullae become roundish, 10 µm thick and connected to neighbor cells by tight junctions at their basal halves (Fig. 3e, f, black arrows). RER becomes more prominent, mitochondria are abundant, and the cytoplasm is enriched in spherical electron-dense bodies of different dimensions (0.5–2 µm) (Fig. 3e, f, red arrows) in the vicinity of which very numerous smaller granules (around 0.5 nm) are also visible (Fig. 3e, f, green arrows).

When the budding nest reaches a dome shape of 1–2 mm in diameter, the number and density of ampullae clearly increase (Figs. 2g, 3g). At this stage, the central vessel and the ramifying ampullae are indistinguishable from each other in histological sections. The epidermal cells change their original round shape and become rather cuboidal, 5–10 µm thick, and in close contact with their neighbors (Fig. 3h). RER, mitochondria, and Golgi become indistinguishable, while numerous of the above-mentioned spherical electron-dense bodies were observed (0.5–2 µm) (Fig. 3h, red arrows) in addition to membrane-free inclusions with heterogeneous content (Fig. 3h, yellow stars) in which remnants of the Golgi apparatus are sometimes visible (Fig. 3i).

Budding nests and dormant spherules have very similar histological and cytological properties (Fig. 3j–l), though the level of differentiation observed during nest formation is even more remarkable in spherules: their cytoplasm is completely filled with dark granules of 2–5 µm and with very amorphous and heterogeneous inclusions (Fig. 3k, l, red arrows and yellow stars, respectively), while smaller granules observed in budding nests (0.5–2 µm) are not visible anymore. The tunic fibers are more compacted (Fig. 3k), potentially conferring the characteristic spherule stiffness.

### Vascular epidermis and mesenchymal cells participate in vasal bud formation

After abscission, the stolonial epidermis and mesenchymal cells contained in the budding nest undergo cellular modifications and morphogenetic movements that eventually lead to the formation of a new individual. We called this process “vasal budding,” and we described the main ontological steps.

#### Step1: swelling

**Fig. 4:**
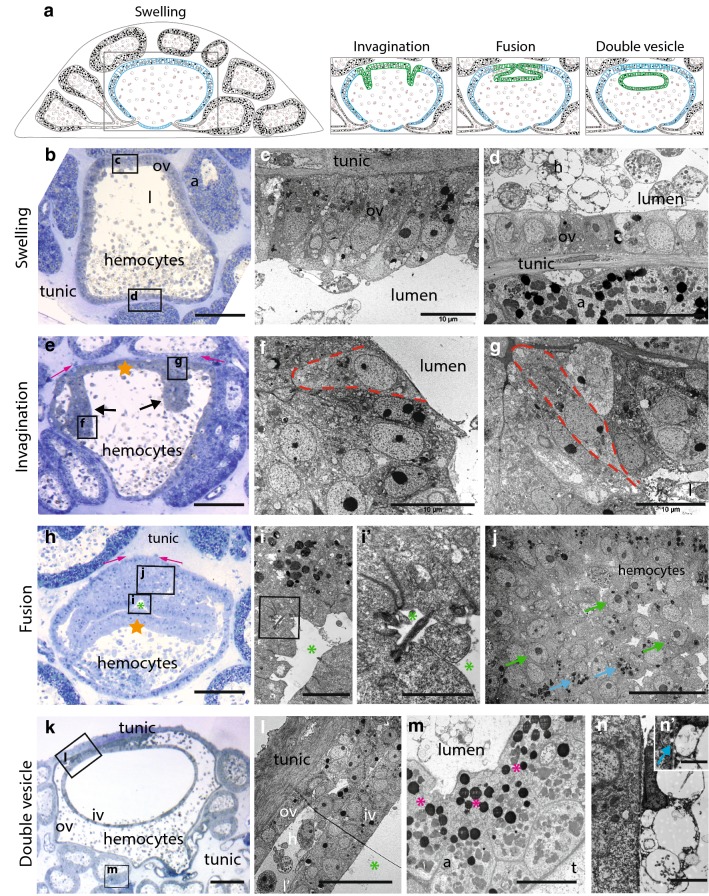
Early stages of budding in *Polyandrocarpa zorritensis*. **a** Schematic summary of vasal budding, depicting the four main steps: swelling, invagination, fusion and double vesicle. **b** Semi-thin section of a vasal bud at the swelling stage. **c** Detail of the vascular epidermis on the top side (squared in **b**). **d** Detail of the vascular epidermis on the bottom side (squared in **b**). **e** Semi-thin section of a vasal bud at the invagination step, showing the invaginating epidermis (black arrows), the movement of the invaginating edges (pink arrows) and the part of the epidermis that goes on to form the floor of the inner vesicle (orange star). **f** Detail of the bottle-shaped cells at the invaginating hinge points (as squared in **e**). **g** Detail of a wedged-shaped cells at the invaginating edge (as squared in **e**). **h** Semi-thin section of a vasal bud at the fusion step, showing the inner vesicle floor (orange star), the inner vesicle lumen (green asterisk) and the movement of the fusing borders (pink arrows). **i** Detailed view of the inner vesicle wall. **i’** Close-up of the cilia apex of the inner vesicle cells (as squared in **i**). **j** Detail of hemocyte aggregate at the fusion area (as squared in **h**), showing undifferentiated hemoblasts (green arrows) and granules-containing cells (blue arrows). **k** Semi-thin section of a vasal bud at the double vesicle stage. **l** Detailed view of hemocytes located between the inner and the outer vesicle. **m** Ampullar wall showing absence of cell membrane and cytoplasmic continuity between cells (pink asterisks). **n–n’** Contact between an ampullar epidermal cell and a morula cell. Blue arrow shows cytoplasmic continuity (Scale bars. **b**, **e**, **h**, **k**: 50 µm; **c**, **f**, **g**, **m**: 10 µm; **d**, **j**, **l** 20 µm; **i**: 5 µm, **i’** 2 µm, **n** 2 µm, **n’** 1 µm) (*a* ampulla, *h* hemocytes, *iv* inner vesicle, *l* vessel lumen, *m* mitochondria, *n* nucleus, *ov* outer vesicle, *t* tunic)

From 0 to 20 h post-abscission (hpa), budding starts by a phenomenon that we called “swelling” (Fig. 4a, b). A more or less spherical cavity about 300 µm in diameter (Fig. 4b) appears at the center of the nest, resulting from inflation of the stolonial vessel. This cavity, which is delimited by a monolayered epithelium, becomes polarized: cells at the top side (i.e., opposite to the substrate) become columnar (approximately 15 µm high × 5 µm wide), while cells at the bottom side (i.e., facing the substrate) are cuboidal (6–8 µm) (Fig. 4c, d, respectively). In comparison with the epithelia of the surrounding ampullae, the cells of the swelling cavity contain few inclusions and a higher nucleo-cytoplasmic ratio (compare *ov* and *a* in Fig. 4d). Heterogeneous populations of mesenchymal hemocytes are visible inside the cavity, inside the connections with ampullae, and inside the ampullae (Fig. 4b,d; Additional file 6: Figure S5a).

#### Step 2: invagination

From 20 to 22 hpa, an epithelial invagination process starts in the swelling vesicle (Fig. 4a, e). At the thicker pole of the cavity (top side), a circular region of the epidermis begins to unfold, which appears as two invaginations on histological sections (Fig. 4e, black arrows). Cells located at hinge points have a typical bottle shape, with their basal surface wider than the apical side (Fig. 4f), while cells at the invaginating edges (Fig. 4g) are wedge-shaped with their basal surface narrower than the apical side. In the course of invagination, the invaginating edges move toward each other (Fig. 4e, pink arrows), thus covering the portion of epithelium located between them (Fig. 4e, orange star).

#### Step 3: fusion

Around 22 hpa, the invaginating edges eventually come into contact and fuse to each other (Fig. 4a, h). This consequently delineates a thin cavity that is the future lumen of the forming inner vesicle (Fig. 4h, i, green asterisk). Cells lining this forming lumen are elongated (20–25 µm high × 3–7 µm wide) and polarized, with clear apical tight junctions as well as an apical cilium (Fig. 4i, i’).

At the point of fusion, the epithelial organization of the invaginating epidermis is lost. A cluster of hemocytes appears around the closing aperture, between the prospective inner and outer vesicle (Fig. 4h, j). The vast majority of the clustering hemocytes show morphological features of undifferentiated hemoblasts, i.e., high nucleo-cytoplasmic ratio, a prominent nucleolus, poorly developed RER and mitochondria (Fig. 4j, green arrowhead), whereas other cells contain few electron-dense granules (Fig. 4J, blue arrowhead). While the basal lamina is present on every epithelial cell of the inner and the outer vesicle (Additional file 6: Figure S5b), it is indiscernible at the fusion point, so that the hemoblast cluster and the invaginating tissue constitute a morphologically indivisible unit without prominent cell junctions.

#### Step 4: double vesicle and initiation of organogenesis

Fusion finally results in the formation of a monolayered inner vesicle, where the apical side of the epithelia faces the lumen of the vesicle, and a monolayered outer vesicle, with the apical side of the epithelium facing the tunic (Fig. 4a, k). Mesenchymal cells remain visible between them, in the region where the fusion occurred (Fig. 4l). While the outer vesicle will give rise to the external epidermis of the new zooid, the inner vesicle folds and undergoes organogenesis to give rise to the different organs of the zooid (Additional file 6: Figure S5c).

From this stage, regression of the surrounding ampullae begins. We have observed significant ultra-structural modifications in the ampullar epidermis, in which nutrients had accumulated during nest formation. At their basal side—facing the lumen—membranes of adjacent epithelial cells lose their integrity and therefore the cytoplasm of all the cells that constitute the ampullar wall is in continuity (Fig. 4m, pink asterisks). In addition, the cytoplasmic electron-dense bodies are displaced toward the basal side of the cells (Fig. 4m). Finally, many mesenchymal cells are in close contact with the ampullar wall (Fig. 4n). These cells contain large and empty vesicles, with a relatively small nucleus and sometimes a nucleolus, corresponding to the classical morphology of morula cells known in other ascidians. At some contact point between these cells and the epidermis, cell membranes are inconspicuous, so that cytoplasmic content of both partners seems to be in continuity (Fig. 4n’, blue arrow).

## Discussion

Among tunicates, solely sexually reproducing species and species that adopt different forms of NED are scattered across the whole subphylum, suggesting that budding and therefore coloniality have been acquired or lost several times [7, 10]. One colonial species in the family Styelidae, *Polyandrocarpa zorritensis,* shows a mode of budding that differs from other family members, which, along with its phylogenetic position [10], reinforces the hypothesis that NED has been acquired convergently in this species.

This paper describes the role of the vascular epidermis and circulating hemocytes in this novel form of NED. We also show that despite the original nature of the budding tissue, bud of *P. zorritensis* convergently acquired some similarities with other tunicate buds, such as the double vesicle stage, the possible participation of hemoblasts, the nutritive role of the epidermis and the existence of a dormant stage. The terms “stolonial budding” and “vascular budding,” which already designate budding modes in other tunicate species such as *Stolonica socialis* and *Botryllus primigenus* [24, 27], are inappropriate for *P. zorritensis.* Both of these budding modes involve cellular sources and early ontogenesis that differ from those we found in *P. zorritensis*. Therefore, given the peculiarities of *P. zorritensis* NED, we propose the term vasal budding.

### Vasal buds originate from a novel combination of tissues

While tunicate budding modes are diverse in terms of tissues involved and early morphogenetic movements [7], most tunicate NED strategies converge morphologically toward a common ontogenetic stage: a triploblastic vesicle formed by two monolayered polarized epithelia that sandwich a mesenchyme of circulating hemocytes [11, 15]. While the outer vesicle always originates from the parental epidermis, the anatomical source of the inner vesicle is highly variable across NED modes. Consequently, budding modes in tunicates have been categorized according to the tissues that give rise to the inner vesicle [16]. For instance, the inner vesicle can originate from: (1) the peribranchial epithelium (e.g., *Botryllus*, *Stolonica*); (2) the epicardial tissue, with or without the participation of the intestinal epithelium (e.g., *Diazona, Aplidium,* and *Polyclinum*); (3) an endostyle outgrowth together with other mesenchymal cells (e.g., *Salpa*, *Pyrosoma,* and *Doliolum*); (4) the stolonial mesenchymal septum (e.g., *Clavelina* and *Perophora*) and (5) the circulating hemoblasts, as in the case of vascular budding (e.g., Subfamily *Botryllinae*) [26, 27, 32]. Here, we describe an additional, yet unreported budding mode that challenges the paradigm that “mature epidermis never gives rise to any tissue other than epidermis itself” [16]. Indeed, in *P. zorritensis*, both the outer and the inner vesicle derive from the parental epidermis through an invagination process. The peculiar onset of *P. zorritensis* vasal budding supports the idea that among ascidians the budding capacity of a tissue may be uncoupled from its embryonic origin.

Studies of two different NED modes in *Botryllus schlosseri*, peribranchial and vascular budding, show that the double vesicle stage exhibits the first spatial segregation of cell fate domains, notably for ectodermal and endodermal identities [33–35]. During vasal budding, the tissue differentiation and the organogenesis also start from the double vesicle stage, wherein organ primordia appears as foldings of the inner vesicle [10]. Therefore, it is possible that, despite the different onset, developmental modules responsible of the zooid morphogenesis are conserved in a convergently evolved NED.

### The role of hemocytes in vasal bud ontogenesis

Formation of vasal buds in *P. zorritensis* involves a coordinated interplay between epithelial and mesenchymal (hemocyte) cells. In several tunicate species, hemocytes are known to participate in bud formation. In particular, undifferentiated cells referred to as hemoblasts (or lymphocyte-like cells), which have been shown to possess multi- or pluripotent stemness capacity, directly contribute to the formation of the inner vesicle [30, 36]. For instance, during stolonial budding of *Perophora*, *Ecteinascidia,* and *Clavelina* the inner vesicle originates from hemoblasts arising from the mesenchymal septum [30, 37, 38], and during Botryllinae vascular budding the circulating hemoblasts give rise to the inner vesicle [27, 32, 39]. Moreover, during peribranchial budding of *Polyandrocarpa misakiensis,* hemocytes appear to integrate into the growing bud by mesenchymal–epithelial transition [40, 41]. In *P. zorritensis* vasal budding, undifferentiated hemoblasts accumulate in the ridge formed by the invaginating epidermis during inner vesicle formation. In this area, the basal lamina is disrupted, the epithelial organization is lost and replaced by a mass of undifferentiated cells. Although based only on morphological data, we hypothesize that hemoblasts integrate into the forming inner vesicle, contributing to its growth and potentially to its differentiation. Studies combining cell proliferation dynamics with molecular markers specific for hemoblasts will be necessary to assay their role in the onset and/or differentiation of the vasal bud. It is unknown so far whether colonial tunicates possess particular population(s) of hemocytes involved in asexual reproduction, which are absent in solitary species. Alternatively, one can speculate that every tunicate possess homologous hemocyte populations, some of them being co-opted for a new function in NED. Morphological and molecular comparative studies on hemocytes at the single cell level would certainly help to solve this intriguing question.

### The role of vascular epidermis and hemocytes in bud nutrition

Among tunicate NED, various populations of differentiated hemocytes function as reserve cells, i.e., they supply nutrients at the onset of budding and during bud growth [25, 42, 43]. Although not functionally tested, this metabolic role of nourishing a bud physically and physiologically separated from the parental zooid has been documented in many species across the three major ascidian taxa, e.g., in trophocyte accumulation in winter stolons of *Clavelina* [44], in strobilated buds of Polyclinidae [16] and in winter buds of *Stolonica socialis* and *Distomus variolosus* [24]. In *P. zorritensis*, formation of the budding nest starts by a thickening of the ampullar epidermis that accumulates lipid and glycogen granules, together with amorphous membrane-free inclusions similar to lysosomal bodies described in other species [45–48]. The lipid nature of the granules is confirmed by their gray color after methylene blue staining (Fig. 4 b) [49] as well as their electron density following osmium fixation (Fig. 3e, f) [50]. Later during bud development, the membranes of these ampullar cells disintegrate, while they are in close contact with numerous hemocytes resembling morula cells [26, 51, 52]. Morula cells have been proposed as cytotoxic cells based on their phenoloxidase activity in different styelid species [53]. Therefore, we suggest that during vasal budding, the energy may be stored in epidermal cells of the ampullae rather than in trophocytes or reserve hemocytes. This is accomplished by glycogen and lipid accumulation and by self-digestion of these epidermal cells that release nutrients to the hemocoel and eventually to the growing buds. Such a nutritive function of the bud epidermis and/or hemocytes has been convergently acquired in species whose buds are physically detached from their parental zooids [24, 25, 31, 45]. Molecular comparison of anabolic and catabolic pathways involved in nutrients accumulation and consumption may shed light on the mechanisms underlying this evolutionary convergence.

### Co-existence of propagating and dormant forms in *P. zorritensis* asexual life cycle

**Fig. 5:**
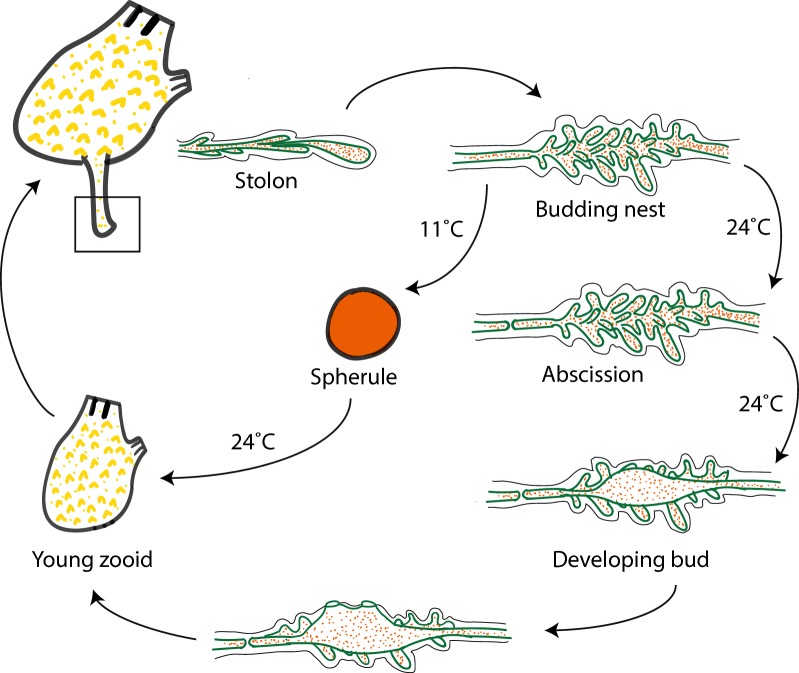
Scheme of the life cycle of *Polyandrocarpa zorritensis*. The adult zooid emits a vascular stolon, along which the ampullae accumulate to form the budding nest. The nest can endure to two different fates: if transferred to low temperature (e.g., 11 °C), it forms a dormant spherule able to develop into a new zooid once transferred to higher temperature; if kept at 24 °C, abscission occurs along the stolon and the nest goes through vasal budding forming a new zooid. In both cases a new zooid able to produce stolons is formed, repeating the cycle and expanding the colony

Previous authors have attempted to classify different tunicate NED modes according to their function within the asexual life cycle of the colony. Mukai et al. [31] distinguished between “buds which are concerned with the growth of the colony,” “buds which are destined to found new colonies,” and “buds whose function is primarily to survive adverse conditions.” The more popular classification by Nakauchi [54] recognized only two categories: “propagative budding”—used for colony growth—and “survival budding”—allowing colonies to survive adverse conditions or damages. To these two categories Turon [45, 55] added “rejuvenative budding” and “multiplicative budding,” respectively, for synchronous regression/renewal cycles and for production of new colonies. In the asexual life cycle of *P. zorritensis*, new zooid can arise either directly from the budding nest or from spherules (Fig. 5). Vasal buds of *P. zorritensis* seem to transcend all of the previously described functional categories, since they play (1) a survival role, in the form of the spherule, which allow for resistance to cold temperatures over winter [56], (2) a propagative role for colonial growth and expansion in the form of budding nest, and potentially (3) a colony-founding role through the dispersal of spherules, which are easily detachable from their mother colony by mechanical action, can be transported by water flow, and eventually allow colony multiplication.

Their behavior and ultrastructure suggests that *P. zorritensis* spherules represent a dormant stage. Dormancy, in general, is a change in physiological state during the life cycle in terms of morphology and metabolism that allows an organism to survive in hostile environments [57], and it appears to have evolved in almost all the groups of colonial animals. Dormancy has not been reported in any tunicate solitary species but it is present in different lineages of colonial ascidians. It is intriguing to speculate that the cellular and molecular mechanisms underlying ascidian NED, such as the stem cells involved in asexual reproduction and regeneration, were shared with dormancy. To our knowledge, *P. zorritensis* is the only known ascidian species where propagative buds and dormant stages coexist at the same time in the same colony. Therefore, *P. zorritensis* represents a striking model to study the relationship between the evolution of NED and the evolution of dormancy.

## Conclusion

In order to understand the evolution of complex traits like non-embryonic development and coloniality, it is necessary to identify suitable, closely related species in which to trace the gains, losses, and modifications of such characters [58]. Subsequently, one must provide detailed descriptions of their NED in order to uncover the cellular and molecular mechanisms that lie behind the budding processes. Due to the heterogeneity and the scattered phylogenetic distribution of NED within the Tunicata, this clade provides an excellent model to take such a comparative approach to understand the origins of NED. A recent phylogenetic analysis of the ascidian family Styelidae [10] highlighted *P. zorritensis* as an amenable model to compare budding mechanisms with NED modes in other more well-studied, closely related, models like in *B. schlosseri* [17, 32] and *P. misakiensis* [27]. The morphological description here forms a foundation for future molecular and cellular studies on the convergent origins on NED within the ascidians.

## Additional files

**Additional file 1: Fig. S1.** Spherules at different degrees of transformation.

**Additional file 2: Fig. S2.** Effect of low-temperature storage on rate of budding and stolon production.

**Additional file 3: Fig. S3.** Relationship between the size of zooids and the size of spherules.

**Additional file 4: Movie 1.** Tip of a stolon with mesenchymal circulating cells.

**Additional file 5: Fig. S4.** Relationship between the size of zooids and the size of budding nests.

**Additional file 6: Fig. S5.** Details of a bud at stage 1, stage 2 and early bud organogenesis.
