# Supplementary material for: Novel budding mode in Polyandrocarpa zorritensis: a model for comparative studies on asexual development and whole body regeneration
Source: EvoDevo. 2019 Apr 3;10:7. doi: 10.1186/s13227-019-0121-x (PMC6446293; doi:10.1186/s13227-019-0121-x)
Supplement: Supplementary file 1 — Additional file 1: Fig. S1. Spherules at different degrees of transformation. [file 13227_2019_121_MOESM1_ESM.pdf]

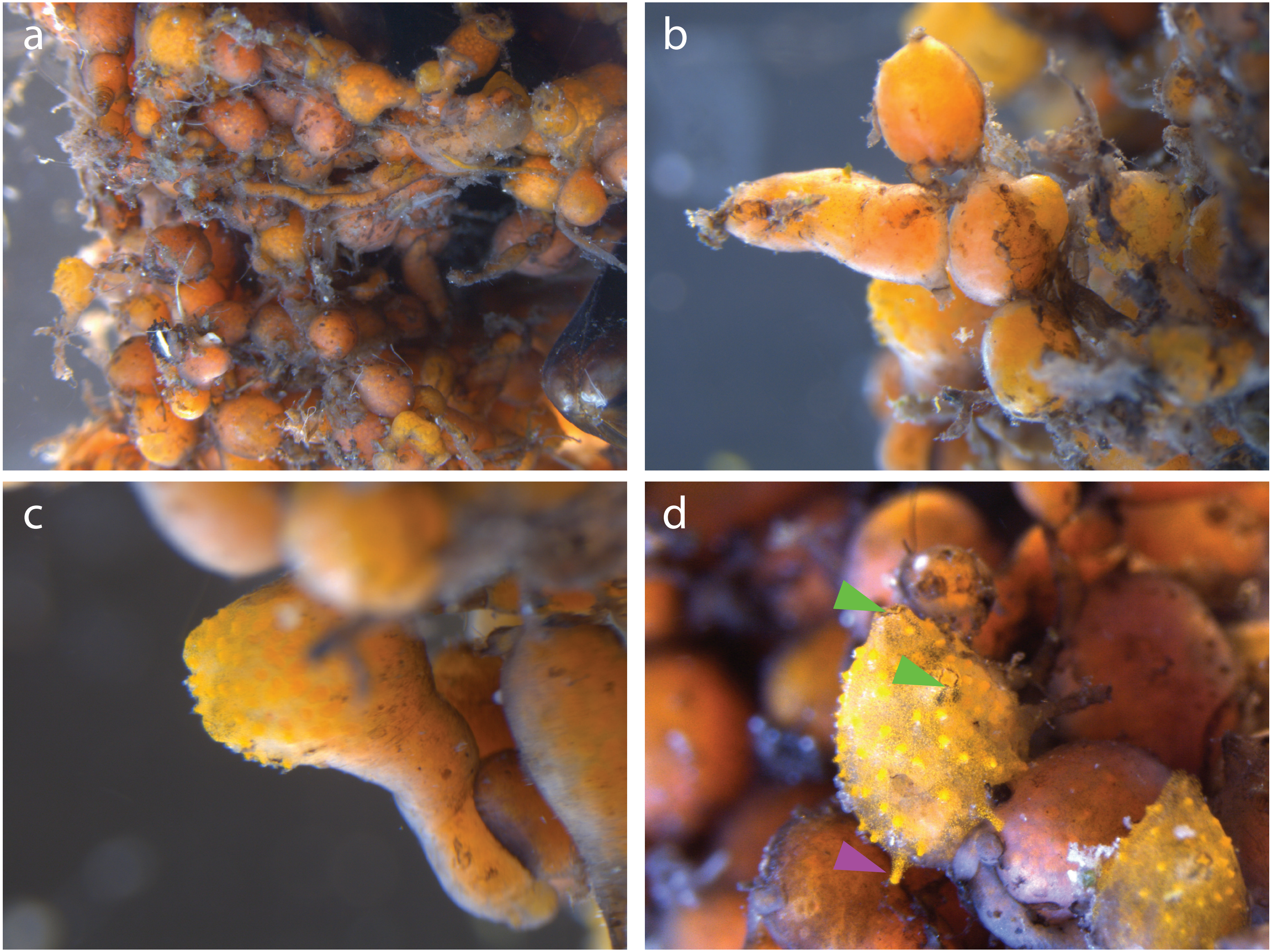

Supp. Fig. 1 Spherules at different degrees of transformation into zooid. a. Bottom af a colony, bearing many spherules at different degrees of transformation. b. An elongated spherule shows early signs of transformation. c. A more advanced stage where yellow pigmentation and rugosity are visible. d. A fully formed zooid, with siphons (green arrowheads) and young stolons (pink arrowhead)
