# Supplementary material for: Novel budding mode in Polyandrocarpa zorritensis: a model for comparative studies on asexual development and whole body regeneration
Source: EvoDevo. 2019 Apr 3;10:7. doi: 10.1186/s13227-019-0121-x (PMC6446293; doi:10.1186/s13227-019-0121-x)
Supplement: Supplementary file 2 — Additional file 2: Fig. S2. Effect of low-temperature storage on rate of budding and stolon production. [file 13227_2019_121_MOESM2_ESM.pdf]

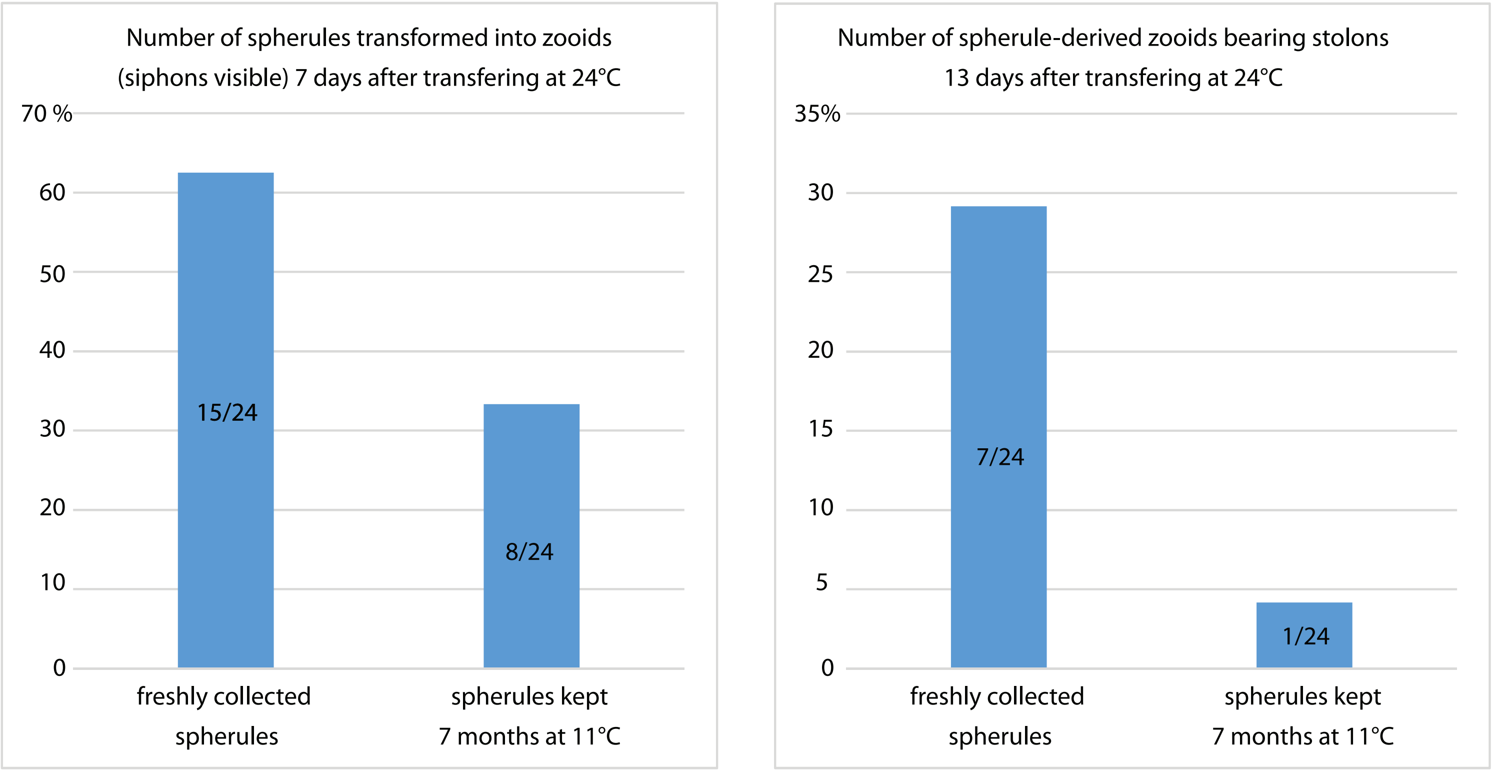

Supp. Fig. 2 Effect of low-temperature storage on rate of budding and stolon production. Left panel. Comparison of the percentage of spherules that have transformed into zooids seven days after being transferred at 24°C, when spherules are freshly collected or kept dormant seven months at 11°C.
Right panel. Comparison of the percentage of spherules that bear stolons thirteen days after being transferred at 24°C, when spherules are freshly collected or kept dormant seven months at 11°C.
