# Supplementary material for: Novel budding mode in Polyandrocarpa zorritensis: a model for comparative studies on asexual development and whole body regeneration
Source: EvoDevo. 2019 Apr 3;10:7. doi: 10.1186/s13227-019-0121-x (PMC6446293; doi:10.1186/s13227-019-0121-x)
Supplement: Supplementary file 3 — Additional file 3: Fig. S3. Relationship between the size of zooids and the size of spherules. [file 13227_2019_121_MOESM3_ESM.pdf]

a

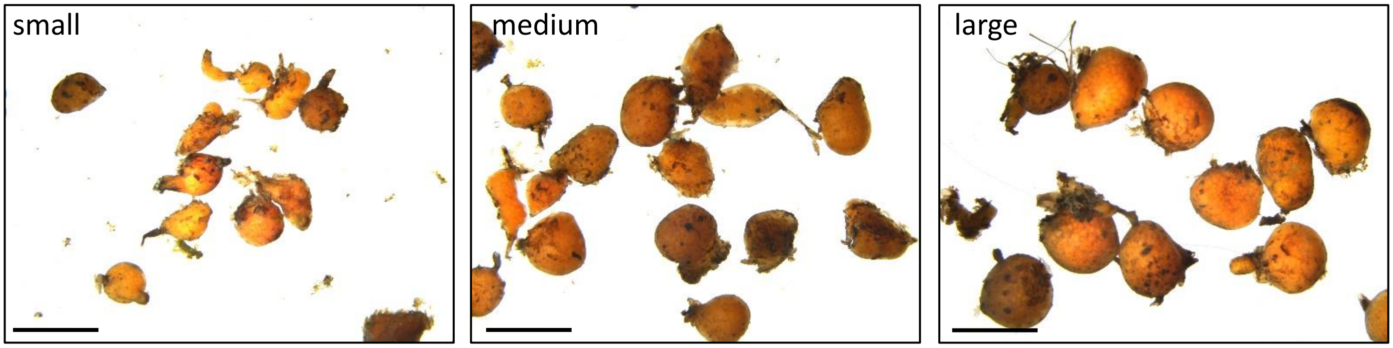

b

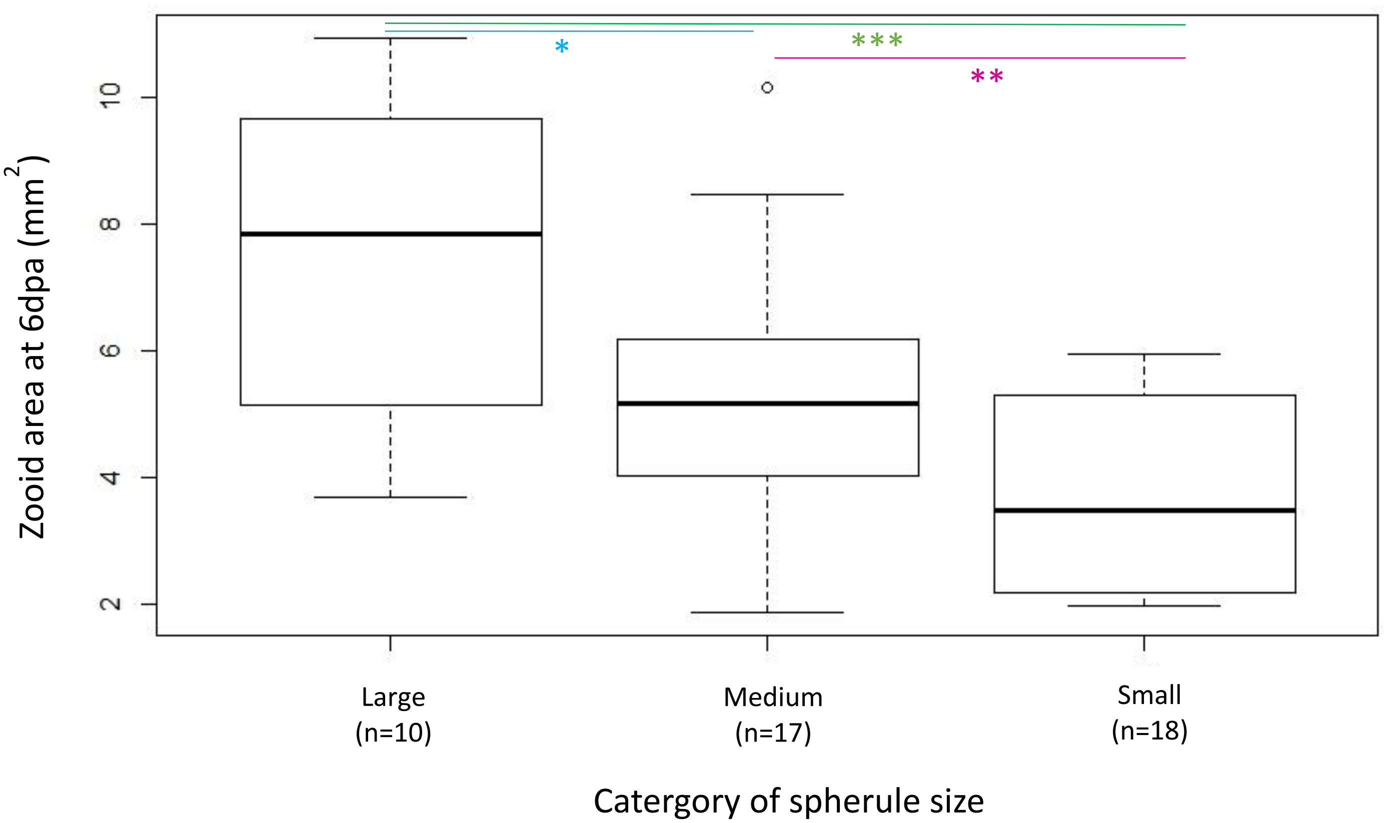

c

	small	medium	large
Number of zooids	21	16	16
Number of stolons	10	18	35
Average number of stolon per zooid	0,5	1,1	2,2

Supp. Fig. 3 **a** Picture of spherules belonging to three categories of size: small, medium, large. Scale bar = 2.5mm. **b** Box-plot showing the relationship between the size of zooids (measured by area) and the size of spherules from which they are formed. **c** Table showing the relationship between the number of stolon per zooid and the size of the spherule.
