# Supplementary material for: Novel budding mode in Polyandrocarpa zorritensis: a model for comparative studies on asexual development and whole body regeneration
Source: EvoDevo. 2019 Apr 3;10:7. doi: 10.1186/s13227-019-0121-x (PMC6446293; doi:10.1186/s13227-019-0121-x)
Supplement: Supplementary file 5 — Additional file 5: Fig. S4. Relationship between the size of zooids and the size of budding nests. [file 13227_2019_121_MOESM5_ESM.pdf]

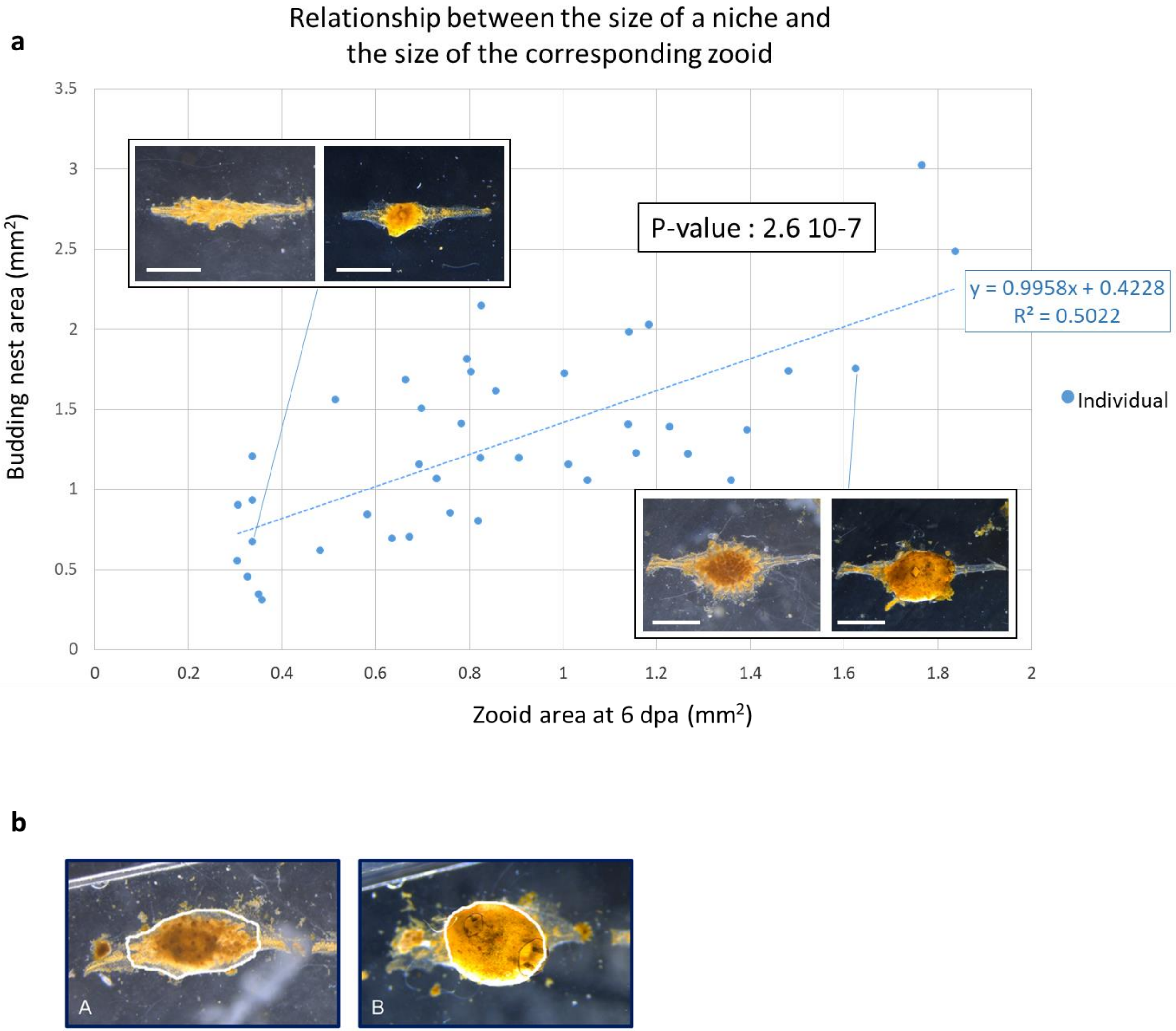

Supp. Fig. 4 **a** Graphical representation of the relationship between the size of a zooid (measured by area) and the size of the budding nest from which it's formed. Scale bar = 1.25mm **b** Example illustrating the tracing of nest and zooids periphery for area measurement
