# Supplementary material for: Novel budding mode in Polyandrocarpa zorritensis: a model for comparative studies on asexual development and whole body regeneration
Source: EvoDevo. 2019 Apr 3;10:7. doi: 10.1186/s13227-019-0121-x (PMC6446293; doi:10.1186/s13227-019-0121-x)
Supplement: Supplementary file 6 — Additional file 6: Fig. S5. Details of a bud at stage 1, stage 2 and early bud organogenesis. [file 13227_2019_121_MOESM6_ESM.pdf]

a

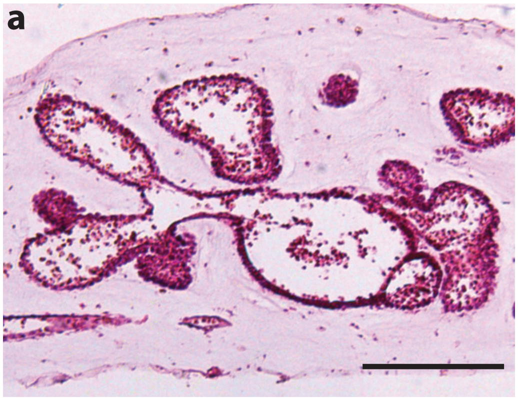

b

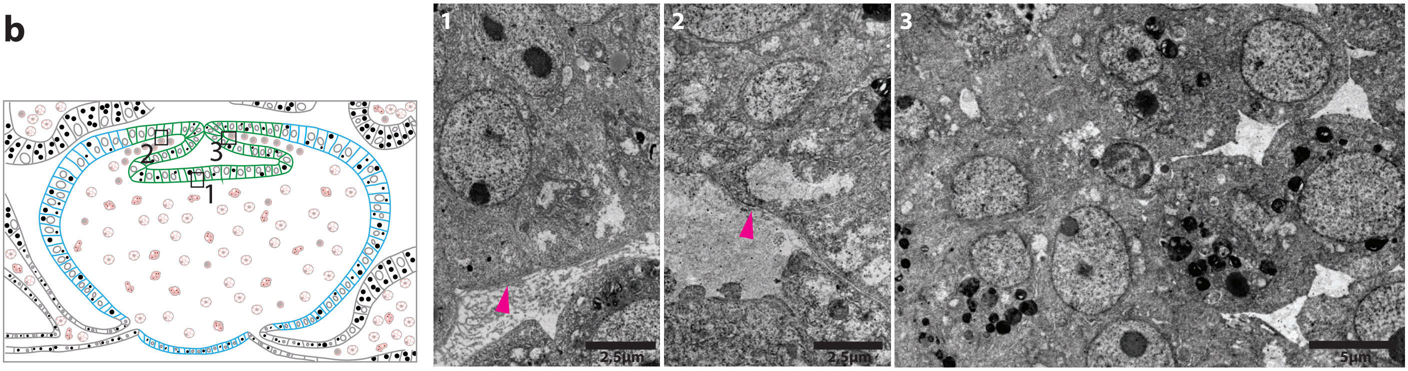

c

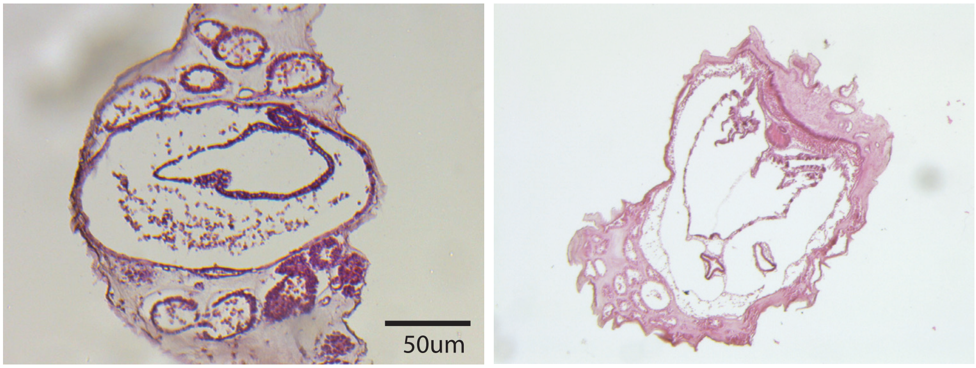

Supp. Fig. 5 **a** Paraffin section (stained with hematoxylin and eosin) showing a bud at the swelling stage and ampullae connected to it. **b** Electron microscopy showing the presence of basal lamina in areas (1) and (2) of the invaginating epidermis and its absence at the point of fusion (3) where hemoblasts cluster. **c** Paraffin sections (stained with hematoxylin and eosin) showing early organogenesis in the inner vesicle (left panel), and later stage where internal organs are visible (right panel)
